# Modern Gynecology

**Published:** 1895-08

**Authors:** 


MODERN GYNECOLOGY. A Treatise on Diseases of Women, comprising the results of the latest investigations and treatment in this branch of medical science. By Charles H. Bushong, M. D. Illustrated. New York: E. B. Treat, No. 5 Cooper Union. 1893. Price, $2.75.

The object of this book may be found in the publisher's preface. We quote for the benefit of our readers:

"This work is designed to fill a place in progressive medicine, and because of its need is here; not as an encyclopædia or manual covering the whole subject, but as a treatise on the *practice of to-day*, 'what to do and how to do it' in the department which it covers."

Turning now to the author's introduction, we quote again:

"The number of books on the diseases of women is already large—so large that it may well be a question if the matter is not already overdone. A glance at the size and pretensions of these works, however, will show that they are all written from one standpoint, that of the specialist. But what of the family physician, of the general practitioner? What is being done for him? This query is especially appropriate for the man who graduated twenty, fifteen, or even ten years ago. When the facilities to-day offered in the best medical colleges for acquiring a knowledge of the diseases of women are compared with those these men had, can we wonder they feel unprepared for this class of work? When they seek to post themselves they are offered a book as large as the volume from which they studied the entire subject of practice of medicine when a student. Is it surprising that busy men should feel their time inadequate for mastering so large a subject? Yet these men are the very ones who should know *well* the essentials of gynecology. To the family physician the women of the family naturally turn as the friend and adviser on whom they can rely. He has been with them during all other forms of illness and is expected to advise them now."

"The effort of this book is to place before the physician a clear common-sense statement of the symptoms of the various diseases of the female sexual organs; to indicate in detail the methods of treatment that can be applied by him, and also to indicate in brief the methods requiring the aid of a specially-trained consultant of larger experience."

These quotations indicate more clearly than any statement we could make what is the value of this candidate for professional favor.

Turning now to the pages of the text we find that the author has done what he promised to do. He has taught all the minute practical points. Prominent among these are the varions steps required to be taken in introducing sounds, pessaries, and other apparatus, all abundantly illustrated. Many a physician will be only too glad to have his ideas cleared up upon the art of placing a pessary. The expert specialist will hardly appreciate it. But then it is not intended for him.

